# Supplementary material for: Should social disconnectedness be included in primary-care screening for cardiometabolic disease? A systematic review of the relationship between everyday stress, social connectedness, and allostatic load
Source: PLoS One. 2019 Dec 19;14(12):e0226717. doi: 10.1371/journal.pone.0226717 (PMC6922387; doi:10.1371/journal.pone.0226717)
Supplement: Attachment — Submitted filename: Response to Reviewers.docx [file pone.0226717.s002.docx]

**Response to Reviewers**

Dear Dr. Kenzie Latham-Mintus,

We thank the editors and reviewers for their valuable comments and suggestions to improve our paper on the link between social connectedness and allostatic load. We have adjusted our manuscript accordingly. Please find below the specific details of how we have addressed each of the editor’s and reviewers’ comments.

**Journal Requirements**

1. **Comment**: When submitting your revision, we need you to address these additional requirements. Please ensure that your manuscript meets PLOS ONE's style requirements, including those for file naming.

**Response**: We have gone through the PLOS ONE formatting guide and adjusted our manuscript to fit the requirements.

2. **Comment**: Please modify the title to identify the work as a systematic review as required by the PRISMA checklist.

**Response**: We have adjusted the title accordingly.

3. **Comment**: Please amend your manuscript to include your abstract after the title page.

**Response**: We have adjusted the manuscript accordingly.

4. **Comment**: Please include your table as part of your main manuscript and remove the individual file. Please note that supplementary tables (should remain/be uploaded) as separate “ supporting information” files.

**Response**: We have inserted the table in the manuscript and removed the individual file.

5. **Comment**: Please include captions for your Supporting Information files at the end of your manuscript, and update any in-text citations to match accordingly. Please see our Supporting Information guidelines for more information.

**Response**: We have no Supporting Information files.

**Reviewer 1 comments**

1. **Comment**: Distill the broad concept of social connectedness to a more workable definition that could be used for screening purposes.

**Response**: We thank the reviewer for highlighting the important issue. We fully agree that our broad conceptualization of social connectedness needs to be addressed in light of the results of our review. We have added text in the Strengths and limitations section on page 27-28, l. 511-520: *“The issue of our broad definition of social connectedness, follows naturally from the previous point. As noted in the methods section, we deliberately accepted an operationalization of social connectedness that included various measures of, for instance, social network size, quality, and content, social support, social activity and integration, etc. Thus, the reviewed studies are somewhat lacking in terms of a clear definition of social connectedness. Nonetheless, consistent with past research, our results strongly indicate that it is both the extent and quality of social connectedness that protect the individual against AL. Hence, in terms of applying social connectedness as a screenable health-risk factor, this suggests that in addition to gauging the mere extent or presence of an individual’s social network, clinical measures should also focus on the quality (e.g. social support, integration, activity) of that network.”*

2. **Comment**: Refine recommendations by devoting more attention to which measurement approaches tended to yield stronger effects.

**Response**: This is an excellent point and we thank the reviewer for bringing our attention to it. We have inserted text in the Strengths and limitations section on page 25, line 452-456: *“In particular, the longitudinal designs employed by Brody et al., Brooks et al., Seeman et al., Weinstein et al., and Yang et al. are noteworthy as strong approaches to the measurement of the link between social connectedness and AL. Most of these studies tapped relatively large and representative populations, often across a decade or more, and as a result generated highly compelling findings and conclusions.”*

**Reviewer 2 comments**

1. **Comment**: Acknowledge the fact that AL indices vary across studies in interpretation of results and as an overall limitation.

**Response**: We thank the reviewer for picking us up on this issue, and absolutely agree that the variability in AL measurement ought to be appropriately acknowledged. We have added text in the Strengths and limitations section on page 27, line 492-496: *“In addition, while the frequent use of multi-component AL measures represents an overall strength in the reviewed research, the cross-study variability in AL indicators may nonetheless complicate efforts to synthesize and compare the results of the different papers. For example, while some studies focused on neuroendocrine markers of AL, others focused on cardiometabolic indices. Future research might mitigate this issue by planning studies around the Allostatic Load Index (ALI) (36) where possible.”*
